# Beyond Blood Sugar: How Left Atrium Strain Predicts Cardiac Outcomes in Type 2 Diabetes

**DOI:** 10.3390/biomedicines12081690

**Published:** 2024-07-29

**Authors:** Laura-Cătălina Benchea, Larisa Anghel, Alexandra Zăvoi, Traian Chiuariu, Silviu-Gabriel Birgoan, Radu Andy Sascău, Cristian Stătescu

**Affiliations:** 1 Internal Medicine Department, “Grigore T. Popa” University of Medicine and Pharmacy, 700503 Iași, Romania; lauracatalina.benchea@gmail.com (L.-C.B.); alexandra.zavoi@gmail.com (A.Z.); traian.chiuariu@gmail.com (T.C.); radu.sascau@umfiasi.ro (R.A.S.); cristian.statescu@umfiasi.ro (C.S.); 2 Cardiology Department, Cardiovascular Diseases Institute “Prof. Dr. George I. M. Georgescu”, 700503 Iași, Romania; birgoan_gabi@yahoo.com

**Keywords:** diabetes mellitus, left atrium strain, cardiac outcomes, heart failure, atrial fibrillation

## Abstract

Speckle tracking echocardiography is an innovative imaging technique that evaluates myocardial motion, including the function of the left atrium (LA). The assessment of the left atrium’s function across its dimensions can have diagnostic and prognostic roles in various cardiovascular conditions. Left atrial strain has been recognized as a valuable predictor of mortality and cardiovascular incidents in the general population across various conditions. For individuals with type 2 diabetes mellitus (T2DM), left atrial dysfunction, as gauged by speckle tracking echocardiography, appears particularly prognostic. Parameters such as peak atrial longitudinal strain (PALS) and left atrial stiffness have been linked with heightened risks of severe cardiovascular events, including atrial fibrillation (AF), heart failure (HF) hospitalizations, or mortality. Consequently, recognizing left atrial dysfunction early is crucial for accurate diagnosis, guiding treatment choices, comprehensive patient management, and prognosis evaluation. Using two-dimensional (2D) speckle tracking echocardiography, results from recent studies report that treatment with empagliflozin significantly enhanced LA function in patients with type 2 diabetes mellitus, improving left atrial strain (LAS) contraction and reservoir values. Furthermore, treatments with glucagon-like peptide-1 (GLP)-1 receptor agonists and sodium–glucose cotransporter-2 (SGLT-2) inhibitors were shown to improve LA reservoir strain more effectively than insulin alone, suggesting their potential in reducing cardiovascular complications in T2DM patients. This narrative review further addresses ongoing challenges and potential enhancements needed to boost the clinical value of left atrium strain, emphasizing its significance in managing and improving outcomes for diabetic patients.

## 1. Introduction

Diabetes mellitus (DM) is a prevalent non-communicable chronic disease, affecting approximately 9.3% of the global population (463 million individuals) as of 2019 [1]. Projections indicate that by 2050, diabetes may affect over 1.31 billion people worldwide [2]. A significant concern is that half of those with diabetes are unaware of their condition, which is particularly alarming given the increased risk of cardiovascular (CV) diseases in these patients [3]. Cardiovascular complications, including coronary artery disease (CAD), stroke, HF, and peripheral artery disease (PAD), are the leading causes of mortality among individuals with diabetes [4]. Type 2 diabetes mellitus (T2DM) accounts for roughly 98% of all diabetes cases, with its prevalence varying significantly across different regions [5]. T2DM is characterized by a complex array of metabolic abnormalities, including insulin resistance, persistent hyperinsulinemia, and hyperglycemia. These conditions lead to alterations in metabolic profiles, intracellular signaling, redox status, and energy production, often resulting in structural and functional changes in the heart before symptoms become apparent. Therefore, early diagnosis and effective management are critical to mitigate the risk of cardiovascular events and reduce mortality associated with this disease. Given the heightened risk of coronary artery disease in diabetic patients, the precision of speckle tracking echocardiography (STE) offers a vital diagnostic tool, enhancing early detection and facilitating timely intervention [6,7]. Stress echocardiography (SE) is effective for diagnosing coronary artery disease and assessing CAD severity. A recent study evaluated the diagnostic value of SE parameters in detecting coronary artery disease severity. Parameters such as global longitudinal strain (GLS), coronary flow velocity reserve in the left-anterior descending artery (CFVR-LAD), and delta wall motion score index (d-WMSI) proved effective, with CFVR-LAD and d-WMSI being particularly useful for identifying severe CAD. Resting GLS demonstrated comparable diagnostic accuracy for CAD detection. These findings support the potential of resting GLS as a screening tool for further angiographic evaluation, pending further validation [8].

**Figure 1:**
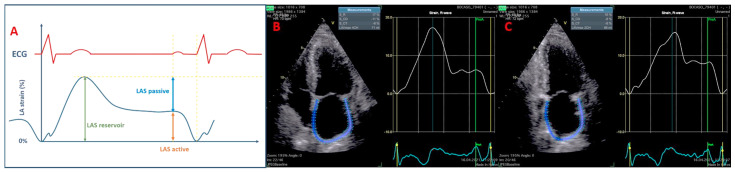
(**A**) The assessment of left atrium function by speckle tracking. (**B**,**C**) LAS in a T2DM patient: reduced LAS reservoir, reduced LAS conduit, and normal LAS pump; LAS reservoir, longitudinal strain during systole; LAS passive (conduit or pool phase), longitudinal strain during early diastole; LAS active (pump phase), longitudinal strain during late diastole; ECG, electrocardiogram; LA, left atrium; LAS, left atrium strain.

LA strain, a novel and efficient echocardiographic tool, evaluates each phase of atrial function: reservoir, pump, and conduit. Specifically, LA reservoir function is gauged by peak atrial longitudinal strain (PALS), pump function by LAS active at the P wave’s onset, and conduit function by LAS passive, which is calculated as PALS minus LAS active (Figure 1). Normal ranges for these functions are 26.1% ± 0.7 for reservoir, 12.0% ± 0.5 for conduit, and 7.7% ± 0.3 for pump function [9].

The use of speckle tracking echocardiography to assess left atrial function offers significant diagnostic and prognostic insights across a range of clinical conditions [10]. This review aims to explore the predictive value of assessing left atrial function in terms of the risk for major adverse cardiovascular events (MACEs), heart failure, atrial fibrillation, and mortality.

## 2. Materials and Methods

Assessing left atrial function via speckle tracking echocardiography is critical for the diagnosis and prognosis of cardiovascular diseases, particularly in patients with type 2 diabetes mellitus. Parameters such as peak atrial longitudinal strain and left atrial stiffness are key predictors of major cardiovascular events like atrial fibrillation, hospitalizations for heart failure, and mortality. Accurate early identification of left atrial dysfunction is imperative for precise diagnosis, guiding therapeutic decisions, and improving clinical outcomes. This review emphasizes the necessity for continued research and advancement to refine the application of left atrial-strain measurements in cardiovascular disease management, especially within diabetic patients. Due to limited existing data, this narrative review was compiled using relevant studies from the PubMed, MEDLINE, and EMBASE databases.

## 3. LA Strain as a Predictor of Cardiovascular Outcomes

Numerous studies have, throughout time, shown that LAS may accurately predict the risk of cardiovascular events and mortality in various patient populations. The average PALS across three apical views was found to be a predictor of cardiovascular morbidity and death in 385 people without AF, HF, or ischemic heart disease [hazard ratio (HR) 1.25, 95% confidence interval (95% CI) 1.09–1.43; *p* = 0.002]. In the general population, PALS predicted the composite outcome for women (HR 1.46, 95% CI 1.05–2.02; *p* = 0.025) and men (HR 0.96, 95% CI 0.81–1.14; *p* = 0.65), which included ischemic heart disease, heart failure, or CV death [11]. According to Bakija F.Z. et al., there is a 2.5-fold increased risk of all-cause mortality for elderly individuals with aberrant PALS values (<32.6%) [12].

In patients with end-stage renal disease (ESRD) receiving hemodialysis or peritoneal dialysis, Ayer A. et al. assessed the relationship between LA speckle tracking echocardiography and the risk of cardiovascular events (a composite of hospitalization for MACE and CV death). Apart from comorbidities, E/e′, and LA volume index (LAVI), lower LA reservoir strain and conduit strain were linked to the primary result (*p* < 0.001) [13].

### 3.1. Heart Failure

Lower PALS (26.4% vs. 36.6%, *p* < 0.001), peak atrial contraction strain (15.6% vs. 16.5%, *p* = 0.016), and LA strain during conduit phase (11.4% vs. 19.3%, *p* < 0.001) at baseline were linked to a higher risk of developing HF in 3.540 individuals from the general population without HF or AF [14].

In a study by Chang C.W. et al., the prognostic value of LA strain was evaluated in 652 individuals, 293 of whom had borderline diastolic function. The ratio of early diastolic trans-mitral flow velocity (E) to early diastolic mitral annular tissue velocity (e′) between 8 and 14 was used to characterize borderline diastolic function. The patients who had a lower than 22.1% LA strain were linked to a higher risk of death from all causes. Patients with an LA strain less than 19.9% had greater chances of CV death. Lower left ventricular (LV) strain was linked to higher CV death in patients with questionable LV diastolic function [15]

An analysis was conducted on 467 patients with sinus rhythm and at least moderate aortic stenosis, to determine the relationship between left atrial strain and a composite of all-cause mortality and hospitalization for HF. The combined end-point (adjusted HR (aHR) 0.95 (95% CI 0.91–0.99), *p* = 0.017) was independently correlated with a PALS score of less than sixteen percent [16].

Moreover, in individuals with acute coronary syndrome (ACS), LAS appears to be a predictor of cardiovascular events. Lower LAS reservoir values (19.6% as the cut-off value) were predictive of the primary outcome of MACE, which included AF, HF, recurrent unstable angina or myocardial infarction (MI), coronary revascularization, nonfatal stroke, and cardiovascular death, in retrospective research including 212 patients with ACS. For a duration of 10.5 ± 2.0 months, the patients were monitored [17].

Rizzetto F. et al. sought to determine the association between atrial strain imaging and cardiovascular outcome in a meta-analysis involving 6021 individuals with HF, valvular heart disease, ischemic heart disease and left ventricular hypertrophy, from nine papers. They came to the conclusion that LAS has an important role in predicting the risk of death or hospitalization due to heart failure [18].

### 3.2. Atrial Fibrillation

PALS and LA contraction strain were also linked to the development of AF, according to a study involving 3590 participants from the fifth Copenhagen City Heart Study. This investigation included subjects with a normal-sized left-atrial and normal LV systolic function [19].

Furthermore, a study by Malagoli et al. evaluated the value of using speckle tracking echocardiography analysis of LA function to predict cardiovascular events (a composite of nonfatal MI, nonfatal stroke, and CV death) in patients with chronic heart failure with reduced ejection fraction (HFrEF). An unfavorable result was independently predicted by lower global PALS assessed at the end of the reservoir phase (hazard ratio, 0.95; 95% CI, 0.94–0.96; *p* = 0.02). Patients with lower PALS had a greater rate of new-onset atrial fibrillation [20].

Following an average follow-up of 49 ± 15 months, patients with lower PALS values (using the discriminatory cut-off value of PALS < 25.5%) had a greater chance of experiencing an episode of AF in a study of 175 patients with chronic COPD and CAD. In order to determine which of these patients are most likely to develop AF, it is critical to evaluate atrial function using speckle tracking echocardiography [21].

During a median follow-up of 16 years, Alhakak A.S. et al. demonstrated, in a study that enrolled 400 individuals from the general population, that PALS is a novel predictor factor for AF and ischemic stroke in participants aged < 65 years [22]. Also, lower values of LA reservoir strain and LA pump strain are associated with a higher risk of AF and a greater frequency of premature atrial complexes and supraventricular tachycardia [23].

Some studies aimed to assess the predictive role of LAS in patients who underwent ablation of long-standing persistent AF. LA contractile strain was the only independent predictor of AF recurrence following ablation [24].

### 3.3. Stroke

In a retrospective study by Park J.H. et al., the predictive value of the global longitudinal strain of LA (LAGLS) was evaluated in 2461 patients with acute HF and sinus rhythm. Patients were followed for an average period of 30.3 ± 25.4 months and the primary outcome was new-onset stroke. During the follow-up, LAGLS was lower in patients with stroke than in those without stroke (14.5% ± 8.8% vs. 17.3% ± 10.5%, *p* = 0.010). The authors concluded that LAGLS with a cut-off value < 14.5% is a good predictor of developing stroke [25].

Also, an impaired LA strain might reflect more advanced atrial cardiomyopathy and might provide a rapid and reliable prognostic risk stratification of acute ischemic stroke (AIS) patients. A recent study evaluated the prognostic significance of left atrial global strain (LA-GSA+) using two-dimensional speckle tracking echocardiography in acute ischemic-stroke patients without atrial fibrillation in an emergency setting. Key findings indicate that reduced LA-GSA+ values are strongly predictive of adverse outcomes, such as death and re-hospitalization, within six months. The study demonstrates that LA-GSA+ can serve as an effective tool for early risk stratification in this patient population, correlating inversely with inflammation markers like C-reactive protein [26].

In a study that encompasses 92 patients with a transient ischemic attack or ischemic stroke, Vera A. et al. aimed to evaluate the association between LAS and stroke recurrence and death. The median follow-up was 28 months, and the primary outcome was a composite of stroke recurrence and death. Lower values of the LAS reservoir and LAS conduit (21% ± 7% vs. 28.8% ± 11%, *p* = 0.017 and 7.7% ± 3.9% vs. 13.7% ± 7%, *p* = 0.007, respectively) were observed among patients who developed the primary outcome. Lower values of the LAS reservoir were associated with a higher risk of stroke recurrence or death after cryptogenic stroke [27].

Also, reduced LA reservoir strain, LA contractile strain, and LA conduit strain were associated with embolic stroke of undetermined source (ESUS) occurrence and AF detection in ESUS patients, suggesting that LA strain assessment may improve risk stratification in ESUS patients [28].

## 4. LA Strain Implications in Diabetes Patients

It has been shown that diabetes patients experience structural alterations and a decline in LA function with time [29]. While diastolic abnormalities are present in 78% of DM patients, LA hypertrophy and dysfunction are unaffected by hypertension or the degree of diastolic dysfunction [30]. Speckle tracking echocardiography plays a crucial role in the clinical management of patients with type 2 diabetes mellitus, by enabling early detection of subclinical cardiac dysfunction. In T2DM, myocardial strain analysis through STE can reveal subtle alterations in myocardial mechanics that are not evident with conventional echocardiography. This is particularly valuable for identifying diabetic cardiomyopathy and assessing the impact of hyperglycemia on cardiac function. By providing detailed insights into global and regional myocardial deformation, STE allows for timely intervention and tailored treatment strategies, potentially improving cardiovascular outcomes in T2DM patients. Consequently, incorporating STE into routine cardiac evaluation for T2DM patients enhances the detection of early myocardial dysfunction and guides more precise clinical decision-making [3,4].

In addition to standard 2D echocardiographic studies, Atas H. et al. used real-time three-dimensional echocardiography (RT3DE) to evaluate the left atrium in 40 diabetic patients and 40 healthy controls. Higher LA minimum (15.6 ± 5.9 vs. 11.9 ± 4.6 mL, *p* = 0.002) and maximal (40.9 ± 11.9 vs. 34.9 ± 9.3 mL, *p* = 0.009) volumes were seen in the diabetes group. In terms of LA function, it was discovered that patients with type 2 DM had decreased pump and reservoir functions (active emptying fraction 38.5 ± 13.0 vs. 46.0 ± 12.4, *p* = 0.007; total emptying fraction: 63.0 (1.5) vs. 66.9 (9.3), *p* = 0.04); there was no difference in the LA conduit function (passive emptying fraction: 38.3 ± 7.4 vs. 36.5 ± 9.9, *p* = 0.36) [29]. Specifically, diabetic patients exhibited increased LA volumes and reduced pump and reservoir functions, highlighting an increased cardiovascular risk and potential for cardiac complications such as atrial fibrillation. These findings emphasize the importance of routine cardiac monitoring in diabetic patients to detect early signs of cardiac dysfunction and to tailor treatment strategies accordingly.

In order to ascertain the predictive significance of left atrial dilatation in individuals with type 2 diabetes mellitus who did not have a known cardiovascular condition, Poulsen conducted a study in 2013. A total of 305 individuals were split into two groups based on their LAVI (LAVI < 32 mL/m^2^ or LAVI > 32 mL/m^2^). A follow-up time of 5.6 years was the mean. Their conclusion was that higher LAVI in T2DM patients was linked to a higher risk of significant adverse cardiovascular events, such as myocardial infarction or ischemic stroke (*p* < 0.001), and a lower overall survival rate (*p* = 0.002) [7]. The correlation between higher LAVI and a lower overall survival rate underscores LAVI’s potential as a prognostic indicator in diabetes management. This suggests that LAVI could be incorporated into routine evaluations to help predict long-term outcomes and potentially guide more aggressive control of diabetes, hypertension, and other modifiable risk factors for those at higher risk.

Diabetic patients bear the burden of microvascular problems resulting from disruptions in microcirculation. In a recent study, Gong et al. assessed the relationship between structural and functional alterations in the left atrium and microvascular complications. The echocardiography was performed in 279 asymptomatic diabetic patients, using 4D-Auto LAQ. Pre-ejection volume, maximum volume, and minimum volume were used to calculate the left atrium’s size. Based on these, the LA channel function, LA storage function, and LA active systolic function were calculated. Microvascular consequences were noted, including peripheral neuropathy, retinopathy, and diabetic nephropathy. The number of microvascular complications (one, two, or three) was directly correlated with an increase in the LAVI [31]. The direct correlation between the number of microvascular complications (including peripheral neuropathy, retinopathy, and diabetic nephropathy) and increased left atrial-volume index underscores the utility of LAVI as a potential marker for assessing the severity of microvascular damage in diabetic patients. This association suggests that changes in the left atrium could reflect broader endothelial dysfunction or microvascular disease burden in diabetes.

In another study, patients with diabetes mellitus (60 patients), prediabetes (50 patients), and healthy controls (60 people) of comparable sex and age had their left atrial function evaluated. The results revealed that LA reservoir and conduit function decreased across the prediabetic and the diabetic patients, while pump function increased [32]. LA reservoir, conduit, and pump functions were assessed in a study that enrolled 134 subjects, including 68 healthy individuals and 66 patients with type 2 DM. The results of this study show that the group of patients with type 2 diabetes had a statistically significant decrease in values for LA reservoir, conduit, and contractile functions (31.2% ± 4.56%, 14.77% ± 6.3%, and 16.36% ± 4.82%, respectively) when compared to the control group (38.75% ± 5.43%, 19.58% ± 5.91%, and 19.16% ± 4.98%, respectively) [33]. The observation that LA reservoir and conduit functions decrease from prediabetic to diabetic states, with an increase in pump function in diabetics, suggests progressive deterioration of LA mechanics as glucose-metabolism disorders worsen. This indicates that even before full-blown diabetes develops, subtle cardiac functional changes are already occurring, highlighting the importance of early detection and intervention.

In another recent study, 155 patients (83 with hypertension, 34 with diabetes, 38 with combined diabetes and hypertension, and 36 matched controls) had speckle tracking echocardiography performed to assess LA function. Compared to controls (39.6 ± 7.8%), PALS was lower in patients with diabetes (24.7 ± 6.4%) and hypertension (29.0 ± 6.5%), and it was even lower in individuals with both conditions (18.3 ± 5.0%) (*p* < 0.0001) [34]. This indicates a correlation between the severity of LA dysfunction and the cumulative burden of diabetes and hypertension. Also, it suggests that the combined effect of these common cardiovascular risk factors on LA function is greater than their individual impacts.

Heart failure with preserved ejection fraction (HFpEF) and T2DM frequently coexist, and this association predisposes the patient to higher mortality and morbidity rates. In 218 patients with heart failure with preserved ejection fraction (HFpEF), including 108 patients with T2DM, PALS, and peak atrial contraction strain were lower in diabetic patients (*p* = 0.002 and *p* = 0.001, respectively). Lower PALS values were significantly associated with a higher prevalence of diabetes mellitus (*p* = 0.002 and *p* = 0.001, respectively), its greater severity (*p* = 0.002 and *p* = 0.016, respectively), and longer duration [35]. The significant association between lower PALS values and the presence, severity, and duration of diabetes in patients with HFpEF indicates that PALS can serve as a valuable prognostic marker. Lower PALS values could potentially be used to identify HFpEF patients at higher risk of adverse outcomes due to the compounded impact of T2DM, thus necessitating more aggressive and tailored therapeutic approaches.

The predictive role of LA strain for cardiovascular events and mortality in people with diabetes has not been well-studied in prior research (Table 1).

PALS and LAVI are strong and independent predictors for the development of new cardiovascular events, such as AF, stroke, transient ischemic attack, MI, coronary revascularization, congestive heart failure, and cardiovascular death, according to research conducted by Cameli M. et al. in a prospective study involving 312 patients with prevalent hypertension (63%) and diabetes (25%) [36]. Zhu S. et al. conducted a prospective study that included 134 individuals with HFpEF and 61 patients with type 2 DM. The group of patients with diabetes mellitus had lower PALS and LAS active (*p* < 0.05). Independent of other clinical and echocardiographic parameters, patients with HFpEF, type 2 DM, and low PALS (<27.2%) had an elevated risk of heart failure hospitalization or death [37]. This highlights the utility of PALS and LAVI in cardiovascular risk stratification, suggesting that these echocardiographic measurements should be routinely utilized in clinical practice to identify high-risk patients early, and to tailor their management accordingly.

Another parameter used to assess LA remodeling is left atrium stiffness (LASt), based on PALS. LASt is calculated using the formula E/e′/PALS [38]. In a study that enrolled 135 patients with HFrEF, including 36 (26.5%) with type 2 diabetes mellitus, Bytyḉi I. et al. analyzed whether LASt can predict cardiovascular events. The primary end-point was a combination of death and hospitalization for HF, and secondary end-points were death and hospitalization. After a follow-up period of 55 ± 37 months, patients with higher values for LASt (cut-off value > 0.82) showed higher rates of cardiovascular events, particularly those with DM [39]. Thus, LASt, particularly when above a cut-off value of 0.82, is a strong predictor of adverse cardiovascular events in patients with HFrEF. The association of higher LASt values with increased cardiovascular events is particularly pronounced in patients with type 2 diabetes mellitus. This suggests that LASt can be an important marker for assessing the severity and progression of heart failure in patients with DM.

In a retrospective study conducted by Arnautu D-M. et al., the authors proposed to verify the hypothesis that left-atrial speckle tracking echocardiography might predict the occurrence of paroxysmal atrial fibrillation in diabetic patients. They concluded that LA strain and LA stiffness are associated with the occurrence of paroxysmal atrial fibrillation in diabetes mellitus [40].

**Table 1:** Studies regarding the predictive role of left atrium strain for cardiac outcomes in type 2-diabetes patients.

Authors	Study Design	Mean Follow-Up	LA Function	Outcomes	Results
**Cameli M. et al.** **(2012)** **[36]**	Prospective, 312 patients, including 25% with T2DM	3.1 ± 1.4 years	PALS cut-off value < −19%	Development of first atrial fibrillation, congestive heart failure, stroke, transient ischemic attack, myocardial infarction, coronary revascularization, and cardiovascular death.	Global PALS is a strong and independent predictor of cardiovascular events and appears to be superior to conventional parameters of LA analysis *p* < 0.0001.
**Zhu S. et al.** **(2022)** **[37]**	Prospective, 164 patients with HFpEF, including 61 (37%) patients with T2DM	13.7 months	Peak LA strain (cut-off value < 27.2%), LA pump function, LA stiffness = E/e’/peak LA strain	Combined outcome of heart-failure hospitalization or death.	HFpEF patients with T2DM and low LAS-peak (HR: 0.93; 95% CI: 0.79–0.90; *p* < 0.001) had a significantly increased risk of heart failure-related hospitalization or death. LAS active (HR: 0.88; 95% CI:0.83–0.94; *p* < 0.001) was significantly predictive of adverse events.
**Arnautu D-A. et al.** **(2023)** **[40]**	Retrospective, 30 adult diabetic patients with documented PAF with 30 age- and sex-matched diabetic patients without PAF		LA global strain, LA-pool strain, LA-pump strain LA stiffness = E/A/peak LA pool strain (cut-off value > 0.48%)	Paroxysmal atrial fibrillation.	The authors found a significant association between reduced LA strains and increased LA stiffness and the presence of PAF in diabetic patients (*p* < 0.05).
**Bytyci I. et al. (2020)** **[39]**	Prospective, 135 patients with HFrEF, including 36 (26.5%) patients with T2DM	55 ± 37 months	LASt = E/e’/PALS (cut-off value > 0.82%)	The primary end-point was a combination of death and hospitalization for HF, and the secondary end-points were death and hospitalization.	Patients with higher values for LASt (cut-off value > 0.82) showed higher rates of cardiovascular events, particularly in those with T2DM (85% sensitivity, 71% specificity and AUC = 0.847, *p* < 0.001).

E/A, the ratio of peak-velocity blood flow from left ventricular relaxation in early diastole (the E wave) to peak-velocity flow in late diastole caused by atrial contraction (the A wave); e′, early diastolic filing; LA, left atrium; LAS, left atrium strain; LASt, left atrium stiffness; HFpEF, heart failure with preserved ejection fraction; HFrEF, heart failure with reduced ejection fraction; PAF, paroxysmal atrial fibrillation; PALS, peak atrial longitudinal strain; T2DM, type 2 diabetes mellitus.

## 5. Clinical Implications and Management

In addition to influencing LV filling pressure, LA plays a crucial role in cardiac function by supporting LV systolic function [41]. LA enlargement and PALS are included in the diastolic-dysfunction diagnostic algorithm. In the assessment of LV filling pressure, missing values are substituted by LA reservoir strain, with a cut-off value of 18% [42]. Diabetes mellitus is a common cardiovascular risk factor that can alter the anatomy and function of the left atrium. The prognostic role of LA function assessment makes it appropriate for use in routine clinical procedures. According to Tadic M. et al., LA function serves as an independent predictor of cardiovascular morbidity and mortality [43]. Two-dimensional speckle tracking echocardiography is a valuable tool for the early detection of atrial dysfunction in patients with HFpEF and T2DM. This technique helps to identify patients who are at high risk of cardiovascular events. Treatment for left atrial dysfunction may start earlier if the condition is diagnosed early. According to the results of a study in which 44 individuals with type 2 diabetes were randomly assigned to receive 10 mg of empagliflozin or a placebo, the medication reduces left atrial strain. Empagliflozin enhanced LA function after a 3-month follow-up period, as measured by increases in the LAS contraction phase and LAS reservoir values (from 10.9 ± 5.7% to 12.5 ± 6.0%; *p* = 0.008) compared with placebo (from 26.4 ± 8.0% to 29.0 ± 7.4%; *p* = 0.011) [44]. Additionally, a different study evaluated how therapy with glucagon-like peptide-1 receptor agonists (GLP-1 RA), SGLT-2 inhibitors, or both, affected left atrial function. A total of 200 T2DM patients receiving metformin treatment were randomly assigned to receive either insulin (50 patients), liraglutide (50 patients), empagliflozin (50 patients), or both (50 patients). Speckle tracking echocardiography was used to quantify left atrial strain, both at baseline and six months after treatment. Patients treated with liraglutide, empagliflozin, or their combination showed improved left atrial reservoir strain (GLP1RA 30.7 ± 9.3 vs. 33.9 ± 9.7%, *p* = 0.011, SGLT2i 30 ± 8.3 vs. 32.3 ± 7.3%, *p* = 0.04, GLP1and SGLT2i 29.1 ± 8.7 vs. 31.3 ± 8.2, *p* = 0.007) as compared to patients treated with insulin (33 ± 8.3% vs. 32.8 ± 7.4, *p* = 0.829) [45]. Furthermore, after receiving dapagliflozin for three months, 61 treatment-naïve patients with type 2 DM who did not have cardiovascular illness showed improvements in their LA reservoir and conduit function [46].

AF is the most common arrhythmia in adults, with a prevalence between 2 and 4%. It is associated with increased mortality and morbidity. The relationship between AF and DM is bidirectional [47]. About 15% of persons with diabetes mellitus develop AF, and about 30% of AF episodes occur in diabetic patients. The identification of some parameters, including echocardiographic ones, which predict the risk of AF in diabetic patients is important, as it presents a series of clinical implications, namely, more frequent 24 h Holter ECG monitoring, diagnosis of subclinical AF, initiation of anticoagulant treatment, and stroke prevention.

**Figure 2:**
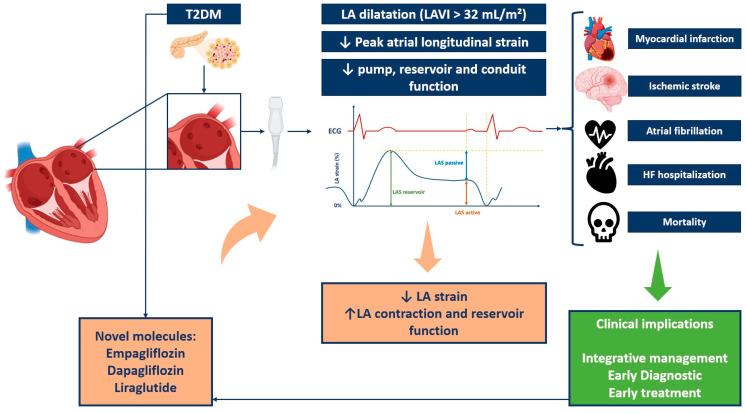
Implications of left atrium strain in type 2 diabetes patients. HF, heart failure; LA, left atrium; LAVI, left atrium volume index; T2DM, type 2 diabetes mellitus.

Subclinical alterations in LA can affect morbidity and mortality in people with diabetes. Speckle tracking-echocardiography assessment of LA function adds new data to the traditional parameters. For this reason, prognostic assessment, integrative management, therapy decision-making, and diagnosis of left atrial dysfunction all depend on an early diagnosis (Figure 2).

## 6. Limitations and Future Directions

In individuals with T2DM, LA dysfunction as assessed by speckle tracking-echocardiography value can offer additional diagnostic and predictive information to traditional echocardiogram parameters. The precise nature of the association between type 2 diabetes and the risk of cardiovascular events remains unclear.

The lack of research relating the effects of interventions on LAS alterations with long-term results is another issue. To evaluate the clinical value of LA function as measured by speckle tracking echocardiography, more extensive multicenter investigations will be needed. Future investigations into LA contractile and conduit functions should be taken into account. The usefulness of LA strain in evaluating the atrial remodeling process and its correlation with thromboembolic risk, AF incidence, and arrhythmia recurrence, has been supported by certain research. In-depth research is required to validate this correlation and determine whether a diagnostic algorithm incorporating LA-dysfunction markers can be applied in routine clinical settings.

Although experts recommend speckle tracking echocardiography to detect subclinical dysfunction, some technical limitations exist [48]. The ability to detect regional function abnormalities differs significantly among vendors, while global strain measurements are highly reproducible. Additionally, the ability to distinguish between scarred and non-scarred segments varies significantly across vendors, with scars in basal segments proving the most challenging to detect [49]. Image quality and standard machine settings (the optimal range of frame rate) seem to be of highest importance for accurate 2D strain estimation. Standard machine settings with a frame rate of 50–60 Hz allow for the correct assessment of peak global longitudinal and circumferential strain. Accurate 2D strain estimation requires correct definition of the region of interest within the myocardium, as well as the reduction in noise and artefacts [50]. Also, the anatomy of each patient, especially the pectus excavatum, can influence the results of speckle tracking echocardiography, potentially leading to misleading interpretations of myocardial function. Notably, left-ventricular functional impairments observed in pectus excavatum individuals often revert after surgical correction of the chest defect, suggesting the alterations may be primarily artifactual rather than intrinsic. Clinicians should consider the degree of anterior chest-wall deformity when interpreting echocardiographic findings in pectus excavatum patients [51].

## 7. Conclusions

Evaluating left atrial function is pivotal for predicting prognosis across various patient groups, including the general population, elderly individuals, and those with clinical conditions such as end-stage renal disease, aortic stenosis, heart failure with reduced ejection fraction, chronic obstructive pulmonary disease, acute coronary syndrome, coronary artery diseases, or borderline diastolic dysfunction. Speckle tracking echocardiography reveals reduced peak atrial longitudinal strain and diminished reservoir function in patients with type 2 diabetes, indicating significant prognostic insights beyond traditional LA echocardiographic assessments. The detection of LA dysfunction, through enhanced left atrial strain and decreased PLAS, can contribute to more effective integrative management strategies, improving patient outcomes.
